# Comparative sequence analysis of the complete set of 40S ribosomal proteins in the Senegalese sole (*Solea senegalensis *Kaup) and Atlantic halibut (*Hippoglossus hippoglossus *L.) (Teleostei: Pleuronectiformes): phylogeny and tissue- and development-specific expression

**DOI:** 10.1186/1471-2148-7-107

**Published:** 2007-07-03

**Authors:** Manuel Manchado, Carlos Infante, Esther Asensio, Jose Pedro Cañavate, Susan E Douglas

**Affiliations:** 1 IFAPA Centro *El Toruño*, Junta de Andalucía Camino Tiro de pichón s/n, 11500 El Puerto de Santa María, Cádiz, Spain; 2 Institute for Marine Biosciences, National Research Council, 1411 Oxford Street, Halifax, Nova Scotia, B3H 3Z1, Canada

## Abstract

**Background:**

Ribosomal proteins (RPs) are key components of ribosomes, the cellular organelle responsible for protein biosynthesis in cells. Their levels can vary as a function of organism growth and development; however, some RPs have been associated with other cellular processes or extraribosomal functions. Their high representation in cDNA libraries has resulted in the increase of RP sequences available from different organisms and their proposal as appropriate molecular markers for phylogenetic analysis.

**Results:**

The development of large-scale genomics of Senegalese sole (*Solea senegalensis*) and Atlantic halibut (*Hippoglossus hippoglossus*), two commercially important flatfish species, has made possible the identification and systematic analysis of the complete set of RP sequences for the small (40S) ribosome subunit. Amino acid sequence comparisons showed a high similarity both between these two flatfish species and with respect to other fish and human. EST analysis revealed the existence of two and four RPS27 genes in Senegalese sole and Atlantic halibut, respectively. Phylogenetic analysis clustered RPS27 in two separate clades with their fish and mammalian counterparts. Steady-state transcript levels for eight RPs (RPS2, RPS3a, RPS15, RPS27-1, RPS27-2, RPS27a, RPS28, and RPS29) in sole were quantitated during larval development and in tissues, using a real-time PCR approach. All eight RPs exhibited different expression patterns in tissues with the lowest levels in brain. On the contrary, RP transcripts increased co-ordinately after first larval feeding reducing progressively during the metamorphic process.

**Conclusion:**

The genomic resources and knowledge developed in this survey will provide new insights into the evolution of Pleuronectiformes. Expression data will contribute to a better understanding of RP functions in fish, especially the mechanisms that govern growth and development in larvae, with implications in aquaculture.

## Background

The eukaryotic ribosome is a complex macromolecular structure composed of a large (60S) and a small (40S) subunit. The large ribosomal subunit catalyses peptide bond formation and is responsible for channelling the nascent proteins through their exit tunnel. The small ribosomal subunit binds mRNA and is responsible for the fidelity of translation by ensuring the correct base pairing between aminoacyl-tRNAs and codons of the mRNA in the decoding centre [1]. Biochemically, the eukaryotic ribosome is composed of four ribosomal RNA molecules and over 70 ribosomal proteins (RPs) [2]. In mammals, the 60S and 40S subunits are composed of 47 and 32 RPs, respectively [3]. Each mammalian RP is typically encoded by a single gene except RPS4 in human [4], and RPS27 in rat [5] and human [6], which are encoded by two separate genes. In contrast, in the yeast *Saccharomyces cerevisiae*, the 78 RPs are encoded by 137 genes, 59 of which are duplicated [7]. In fish, the complete set of RPs in *Fugu rubripes *[8] and *Ictalurus punctatus *[9] has been described. Of the 32 RPs from the 40S subunit, a duplication of RPS27 in both species and of RPS26 in *I. punctatus *was observed. In the 47 RPs from the 60S subunit, all of them but one (RPL5 in *I. punctatus*) appeared to have only one type of mRNA [10].

RPs play a critical role in protein biosynthesis. Cellular levels change as a function of growth rate in bacteria and fungi [11-14]. In fish, mRNA levels increase co-ordinately during embryogenesis and larval development [15-18]. In mammals, certain tumors have substantially increased levels of some RP transcripts [19,20]. However, different RPs have also been associated with various other cellular processes; the so-called extraribosomal functions. For example, in Drosophila, mutations in the RPS2 gene appear to cause arrest of oogenesis [21] and RPS6 functions as a tumor suppressor in the hematopoietic system [22]. Mammalian RPS3 appears to possess apurinic/apyrimidinic endonuclease activity involved in DNA repair functions [23]. Haploinsufficiency of the RPS4 genes has been suggested to contribute to anatomic abnormalities associated with the Turner syndrome in humans [4]. The gene encoding RPS19 seems to participate in embryogenesis due to its capacity to interact with FGF-2, a factor involved in the differentiation process of different cell types [24]. Finally, apoptosis can be induced by inhibiting or activating expression of RPS3a and RPS27L, respectively [6,25].

Senegalese sole, *Solea senegalensis *(Pleuronectiformes: Soleidae), and Atlantic halibut, *Hippoglossus hippoglossus *(Pleuronectiformes: Pleuronectidae), are two commercially important flatfish species. During larval development, both species change from a symmetrical morphology to an asymmetric, benthic juvenile. This metamorphic process involves dramatic morphological and physiological changes. In Senegalese sole, metamorphosis occurs very early during larval development, between 12 and 19 days after hatching (DAH) [26]. In Atlantic halibut, metamorphosis begins with the migration of the left eye about 80 DAH [27]. Apoptotic processes induced by thyroxine hormone have been associated with this tissue remodelling in flatfish [28]. In addition, Senegalese sole larvae exhibit two different growth rates during development [26,29]. Because of the key role RPs play in cellular growth and proliferation and in some cases apoptosis, it is important to elucidate the expression pattern of RPs during flatfish development.

RPs are highly represented in cDNA libraries [9,10]. The development of large-scale genomics on Senegalese sole and Atlantic halibut has made possible an efficient and systematic analysis of RP sequences in both species. In this work, we report the complete set of 32 40S subunit RP cDNAs for both Senegalese sole and Atlantic halibut and describe their main characteristics. Comparative sequence analysis revealed the existence of two and four RPS27 genes in Senegalese sole and Atlantic halibut, respectively. Real-time PCR analysis revealed different RP expression patterns during larval development and in tissues in sole.

## Results

### Characteristics of the 40S RPs

Sequence analysis of normalized libraries for Senegalese sole allowed the identification of 31 out of 32 40S subunit RPs (only RPS28 was absent). RPS28 was obtained from a premetamorphic stage larval library using specific primers (Table 1). Overall, 40S RP genes were not highly represented in the normalized libraries accounting for 252 (2.5%) out of the 10,099 good sequences. The number of clones for each RP ranged between 24 for RPS2 and only 1 for RPS27-2 and RPS29 (Table 2A).

**Table 1:** Primers used for real-time PCR gene expression analysis. F and R refer to forward and reverse primers, respectively.

Target	Primers			Fragment size (bp)
		
	Primer pair name	Sequence	5'-position	
RPS2	SserpS2•1	5'-CCAAGCTGTCGATTGTCCCGGTCA-3' (F)	434	127
	SserpS2•2	5'-CGGGGGGCAGGGATGAGACG-3' (R)	560	
RPS3a	SserpS3a•1	5'-TCAGAAAGACCTCCTACGCCCAGCA-3' (F)	450	94
	SserpS3a•2	5'-AGATCATTGGTCTGAACCTCACGGGTCA-3' (R)	543	
RPS15	SserpS15•1	5'-CATGGTTGGCGTGTACAATGGCAAA-3' (F)	300	116
	SserpS15•2	5'-GGCGACCGTGCTTGACTGGCTTG-3'(R)	415	
RPS27-1	SserpS27-1•1	5'-CCCGAGGAGGAGAAGAGGAGGCACA-3' (F)	64	124
	SserpS27-1•2	5'-CTGTCTGAGCGTGACTGAACACCGTCGT-3' (R)	187	
RPS27-2	SserpS27-2•1	5'-GCTAAAGACCTCCTCCACCCTGCCATT-3' (F)	72	130
	SserpS27-2•2	5'-ACACAGTTGTGATTTTGTAGCAGCCTGGAC-3' (R)	201	
RPS27a	SserpS27a•1	5'-GCGTGAGTGTCCGGCTGACGA-3' (F)	431	87
	SserpS27a•2	5'-GTGAGGCAGCACTTCCCGCAGT-3' (R)	517	
RPS28	SserpS28•1	5'-CGATAGTTCCCGCTGAAGCTGTGAGGTG-3' (F)	206	95
	SserpS28•2	5'-GAGAATGTGAGGGATGTCCGCCGTTG-3' (R)	300	
RPS29	SserpS29•1	5'-AGGCAGTACGCTAAAGACATCGGCTTCGTG-3' (F)	135	121
	SserpS29•2	5'-GTGCTGAATTATCCCATCATCTTGGCTGGT-3' (R)	255	
GAPDH	SseGAPDH231•1	5'-AGCCACCGTGTCGCCGACCT-3' (F)	1001	107
	SseGAPDH231•2	5'-AAAAGAGGAGATGGTGGGGGGTGGT-3' (R)	1107	
Ubiquitin	SseUB•1	5'-AGCTGGCCCAGAAATATAACTGCGACA-3' (F)	289	93
	SseUB•2	5'-ACTTCTTCTTGCGGCAGTTGACAGCAC-3' (R)	381	

**Table 2:** Structural characteristics of the cDNAs encoding RPs of (A) Senegalese sole, *Solea senegalensis *and (B) Atlantic halibut, *Hippoglossus hippoglossus*. Lengths of coding regions, available 5'-UTR, 3'-UTR and poly (A) tail distances from poly(A) signals are indicated. Asterisk (*) denotes RPS coding sequences that were derived partially or completely from sequences present in GenBank. ND, not detected.

(A)						
Gene	# clones	Accession #	Coding region	5'-UTR	3'-UTR	Poly(A) from poly(A)Signal

RPSa	15	AB291586	942	60	41	17
RPS2	24	AB291554	843	ND	39	12
RPS3	16	AB291555	738	9	84	17
RPS3a	13	AB291556	801	1	41	17
RPS4	7	AB291557	792	ND	57	17
RPS5	5	AB291558	612	29	62	37
RPS6	14	AB291559	750	23	39	22
RPS7	19	AB291560	585	51	36	13
RPS8	6	AB291561	627	ND	52	24
RPS9	6	AB291562	585	46	67	14
RPS10	9	AB291563	501	5	44	15
RPS11	7	AB291564	486	11	77	26
RPS12	16	AB291565	399	54	23	11
RPS13	4	AB291566	456	3	37	13
RPS14	2	AB291567	456	37	43	13
RPS15	4	AB291568	438	24	43	16
RPS15a	4	AB291569	393	34	38	16
RPS16	3	AB291570	441	18	39	14
RPS17	2	AB291571	405	21	39	11
RPS18	10	AB291572	459	5	39	15
RPS19	7	AB291573	444	5	22	22
RPS20	6	AB291574	360	98	54	24, 29
RPS21	4	AB291575	252	92	44	7
RPS23	5	AB291576	451	19	41	14
RPS24	9	AB291577	396	17	84	22
RPS25	5	AB291578	372	6	66	24
RPS26	6	AB291579	348	23	47	15
RPS27-1	7	AB291580	255	30	200	20
RPS27-2	1	AB291581	255	62	190	13
RPS27a	7	AB291582	471	77	36	19
RPS28	-	AB291583	210	1	126	21
RPS29	1	AB291584	171	5	103	14
RPS30	8	AB291585	402	35	96	15

(B)						

Gene	# clones	Accession #	Coding region	5'-UTR	3'-UTR	Poly(A) from poly(A) Signal

RPSa	3	EB034722, EB039353	930	84	41	17
RPS2*	4	EB029719, EB030051	858	3	41	12
RPS3	2	EB034826, EB036030	741	25	126	12
RPS3a	6	EB035413, EB031090	801	23	41	14
RPS4	8	EB032598	792	41	57	20
RPS5	2	EB032359 EB036495	612	44	44	14
RPS6	2	EB031692, EB032095	750	40	33	14
RPS7	7	EB032431	585	37	33	14
RPS8	5	EB035663	627	24	39	11
RPS9	2	EB040140, EB032308	585	46	60	19
RPS10	2	EB031080	501	39	41	15
RPS11	5	EB035513	486	23	67	11
RPS12	1	EB029693	399	65	23	11
RPS13	1	EB034224	456	21	41	14
RPS14	3	EB040078	456	43	38	12
RPS15	3	EB036829, EB029855	438	25	42	18
RPS15a	2	EB036744	393	212	36	14
RPS16	1	EB038832	441	33	44	19
RPS17	1	EB035783	405	24	38	12
RPS18	6	EB035452	459	25	46	14
RPS19	2	EB030274	444	27	21	20
RPS20	2	EB038732	360	97	34	12
RPS21	3	EB030651	252	71	45	8
RPS23	5	EB035807	432	59	42	16, 21
RPS24	1	EB034880	399	34	81	19
RPS25	3	EB040606, EB040657	396	21	49	12
RPS26	7	EB036835	348	29	49	11
RPS27-1	4	EB039502	255	73	207	11
RPS27-2*		DN794622	255	52	192	8
RPS27-3	5	EB039943	255	73	>635	>451
RPS27-4	1	EB040256	249	84	>402	>74
RPS27a	4	EB038389	471	52	43	17
RPS28	1	EB037921	210	22	123	16
RPS29*		DN792676	171	30	>103	>15
RPS30	4	EB032882	402	59	96	15

* GenBank sequences comprise final contig

Gene sizes for the complete set of 40S RPs ranged between 279 and 1,043 bp for RPS29 and RPSa, respectively. Only RPS2, RPS4 and RPS8 had partial sequences missing the 5'-ends. All cDNA sequences have been deposited in the GenBank/EMBL/DDBJ with accession numbers from AB291554 to AB291586 (Table 2A). Most RPs (63.6%) used TAA as termination codon. Only RPS8, RPS11, RPS12, RPS15, RPS17, RPS27-1, RPS28, and RPS29 used TAG, and RPS6 and RPS24, TGA. The 3'-UTRs were highly AT-rich. All RPs had a canonical AATAAA polyadenylation signal between 7–37 nucleotides from the poly(A) tail.

In halibut, sequences for all except RPS29 and RPS27-2 were identified from the Pleurogene database (Table 2B). In most cases, the complete coding sequences were obtained, but 3'-end sequencing was performed for all RP sequences to confirm the 3'ends, particularly of the long ESTs. The RPS29 and RPS27-2 sequences presented in this analysis derive from Atlantic halibut ESTs in GenBank [GenBank:DN792676, GenBank:DN794622]. In addition, only a partial sequence for RPS2 was obtained and the 5'-end was completed by the addition of an Atlantic halibut EST in GenBank [GenBank:CF931586]. 108 ESTs from a total of 12,675 sequences in the database encoded RPs (0.9%), an indication of the excellent normalization in these cDNA libraries.

Sizes of coding sequences, 5'- and 3'-UTRs and positions of polyadenylation signals are given in Table 2B. All RPs had a single polyadenylation signal with the exception of RPS20, which had two possible non-canonical sites (AtTAAA and AATgAA), and RPS23, which had two possible canonical sites. The polyadenylation signal for RPS19 overlapped the stop signal and no polyadenylation signal could be identified in RPS27-3. As with Senegalese sole, most 40S subunit RPs used TAA (59%) as a stop codon, followed by TAG (26%) and TGA (15%) and the 3'-UTRs were very AT-rich. Two ESTs comprising RPS14 and RPS27a contigs differed in the lengths of their 5'-UTRs by over 100 nucleotides due to the presence of unspliced introns in this region.

### Comparison of the Senegalese sole and Atlantic halibut 40S subunit RPs

The complete set of 40S RPs in Senegalese sole and Atlantic halibut had a high overall similarity (92.1%) as determined by deduced amino acid sequences (Table 3). With respect to the other species, Senegalese sole overall similarities ranged between 92.8 and 95.4% with human and *F. rubripes*, respectively. For halibut, these values were 89.8 and 92.5% with human and *F. rubripes*, respectively. The most conserved 40S RPs in the five species (overall mean >99%) were RPS23 and RPS27a; and the most divergent ones were RPS30 (86.9%), RPS25 (87.9%), and RPSa (89.0%).

**Table 3:** Amino acid comparisons of the RPs from *S. senegalensis *(Sse) and *H. hippoglossus *(Hhi) with those of *I. punctatus *(Ipu), *F. rubripes *(Fru) and human (Hsa). Similarity values for Senegalese sole and Atlantic halibut are separated by "/".

	Number of aminoacids					Similarity (%)			
		
Gene	*Sse*	*Hhi*	*Ipu*	*Fru*	*Hsa*	*Sse/Hhi*	*Ipu*	*Fru*	*Hsa*
Sa	313	309	317	306	295	91.3	88.5/88.0	90.5/89.5	86.8/88.1
S2	280	285	278	279	293	94.0	96.3/91.0	97.3/92.0	90.0/85.0
S3	245	246	245	245	243	97.6	98.8/96.3	99.2/96.7	95.9/96.3
S3a	266	266	266	266	264	96.6	94.7/94.7	96.2/95.5	93.9/93.6
S4	263	263	263	263	263 263	96.6	95.8/93.9	95.8/94.3	93.5/92.8^a^ 89.4/88.2^b^
S5	203	203	203	203	204	99.5	99.0/98.5	97.0/96.6	97.5/97.0
S6	249	249	249	249	249	98.4	95.6/94.0	98.4/97.2	95.6/94.4
S7	194	194	194	194	194	98.5	96.4/95.9	98.5/99.0	96.9/96.4
S8	208	208	208	208	208	98.1	89.4/88.9	96.2/94.2	94.2/93.8
S9	194	194	194	194	194	97.9	95.9/96.9	97.4/96.6	95.9/96.9
S10	166	166	166	166	165	97.6	96.4/94.6	97.0/95.8	90.3/89.7
S11	161	161	159	161	158	98.1	90.6/91.8	97.5/96.9	89.2/89.2
S12	132	132	132	132	132	97.7	97.7/95.5	98.5/97.7	97.7/96.2
S13	151	151	151	151	151	98.0	98.7/98.0	100/98.0	98.7/96.7
S14	151	151	151	151	151	96.0	95.4/99.3	96.0/100	96.0/100
S15	145	145	145	145	145	98.6	96.6/96.6	99.3/99.3	94.5/94.5
S15a	130	130	130	130	130	100	96.9/96.9	99.2/99.2	97.7/97.7
S16	146	146	146	146	146	97.9	96.6/97.3	95.2/95.2	96.6/95.9
S17	134	134	134	134	135	92.5	92.5/96.3	91.8/97.0	92.5/96.3
S18	152	152	152	152	152	98.7	99.3/98.0	100/98.7	98.0/98.0
S19	147	147	147	146	145	92.5	89.1/87.1	95.2/95.9	86.9/84.1
S20	119	119	119	119	119	98.3	98.3/97.5	100/98.3	98.3/97.5
S21	83	83	83	83	83	92.8	92.8/90.4	95.2/89.2	94.0/95.2
S23	143	143	143	143	143	100	99.3/99.3	100/100	98.6/98.6
S24	131	132	131	131	133	98.5	96.9/96.2	95.4/97.7	90.1/90.9
S25	123	131	124	123	125	95.1	83.1/82.3	96.7/95.1	82.4/80.8
S26	115	115	115	115	115	98.3	93.0/94.8^c^ 93.9/94.8^d^	96.5/98.3	93.9/95.7
S27-1	84	84	84	84	84	See Table 4			
S27-2	84	84	84	84	84				
S27-3		84							
S27-4		82							
S27a	156	156	156	156	156	100	100/100	100/100	98.7/98.7
S28	69	69	69	69	69	98.6	100/98.6	97.1/95.7	95.7/94.2
S29	56	56	56	56	56	98.2	100/98.2	100/98.2	98.2/96.4
S30	133	133	133	133	133	94.7	85.8/85.0	90.3/91.0	80.6/81.2
Overall						92.1	94.0/90.9	95.4/92.5	92.8/89.8

^a ^and ^b ^refer to similarities to *H. sapiens *RPS4X and RPS4Y isoforms, respectively.

^c ^and ^d ^refer to similarities to *I. punctatus *RPS26-1 and RPS26-2 isoforms, respectively.

The number of amino acids was highly conserved throughout evolution. Of the 32 40S subunit RP cDNAs, 21 had ORFs with identical number of amino acids in the five species compared; RPS3 and RPS24 showed different sizes in Atlantic halibut and human. Four RPs had the same number of amino acids among fish species but different from human (RPS3a, RPS5, RPS10, and RPS17). In addition, three others varied in more than two species (RPSa, RPS2, and RPS25) (Table 3). Senegalese sole RPSa and RPS2 showed the highest differences in size being 18 amino acids longer and 13 amino acids shorter than in human, respectively.

### Phylogenetic analysis of RPS27 genes

**Figure 1:**
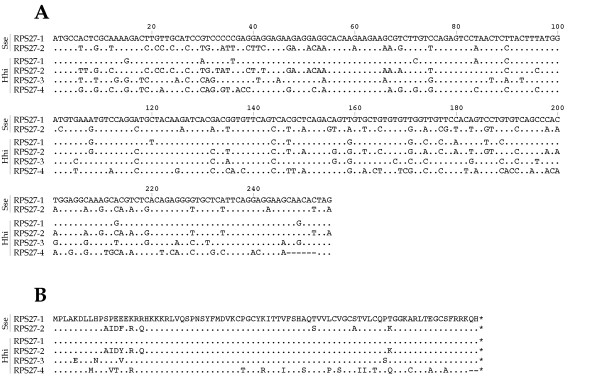
RPS27 genes from *S. senegalensis *(Sse) and *H. hippoglossus *(Hhi). (A) Coding sequence alignment. (B) Amino acid alignment. Dots indicate identity and hyphens represent indels.

Two and four cDNAs encoding RPS27 were found in Senegalese sole (referred to as RPS27-1 and RPS27-2) and Atlantic halibut (referred to as RPS27-1, RPS27-2, RPS27-3, and RPS27-4), respectively (Figure 1A). In Senegalese sole, the total lengths of RPS27-1 and RPS27-2 were 485 and 507 nucleotides (nt), respectively. They were represented by 7 and 1 clones, respectively (Table 2A). In Atlantic halibut, total lengths ranged between 499 and 963 nt for RPS27-2 and RPS27-3, respectively. The Atlantic halibut RPS27-4 had a slightly shorter coding region (249 nt), whereas no putative polyadenylation signal could be identified in RPS27-3. RPS27-1 and RPS27-3 were represented by 4 and 5 clones in the halibut libraries, respectively, and RPS27-4 by only 1 EST.

Both Senegalese sole paralogs showed a high divergence at the nucleotide level when complete cDNAs (48.4% identity) or coding regions (73.7%) were aligned. At the amino acid level, they differed in 9 residues with a sequence identity of 89.3% (Figure 1B; Table 4). Similarly, low sequence similarity (36.5–49.4%) was detected among Atlantic halibut full-length sequences. These values ranged between 66.7 (RPS27-1 and RPS27-4) and 82.0% (RPS27-1 and RPS27-3) in the coding regions. At the protein level, RPS27-4 was 2 amino acids shorter that the other paralogs. Amino acid similarities ranged between 74.4 (RPS27-3 and RPS27-4 with 21 amino acid changes) and 95.2% (RPS27-1 and RPS27-3 with 4 residue differences). Among species, *S. senegalensis *RPS27-1 and *H. hippoglossus *RPS27-1 were the closest evolutionary homologues with 82.5% similarity using full-length sequence, 92.5% in coding sequence and 100% in amino acid sequence (Table 4).

**Table 4:** Amino acid similarities of RPS27 from *S. senegalensis *(Sse) and *H. hippoglossus *(Hhi) with those of *I. punctatus *(Ipu), *F. rubripes *(Fru) and *H. sapiens *(Hsa).

	Sse RPS27-2	Hhi RPS27-1	Hhi RPS27-2	Hhi RPS27-3	Hhi RPS27-4	Ipu RPS27-1	Ipu RPS27-2	Fru RPS27-1	Hsa RPS27-1	Hsa RPS27-L
SseRPS27-1	89.3	100.0	91.7	95.2	79.3	96.4	97.6	100.0	98.8	95.2
SseRPS27-2		89.3	96.4	85.7	74.4	90.5	90.5	89.3	89.3	86.9
HhiRPS27-1			91.7	95.2	79.3	96.4	97.6	100.0	98.8	95.2
HhiRPS27-2				88.1	76.8	92.9	92.9	91.7	91.7	89.3
HhiRPS27-3					74.4	91.7	92.9	95.2	94.0	91.7
HhiRPS27-4						79.3	79.3	79.3	78.0	75.6
IpuRPS27-1							98.8	96.4	95.2	92.9
IpuRPS27-2								97.6	96.4	92.9
FruRPS27-1									98.8	95.2
HsaRPS27-1										96.4

**Figure 2:**
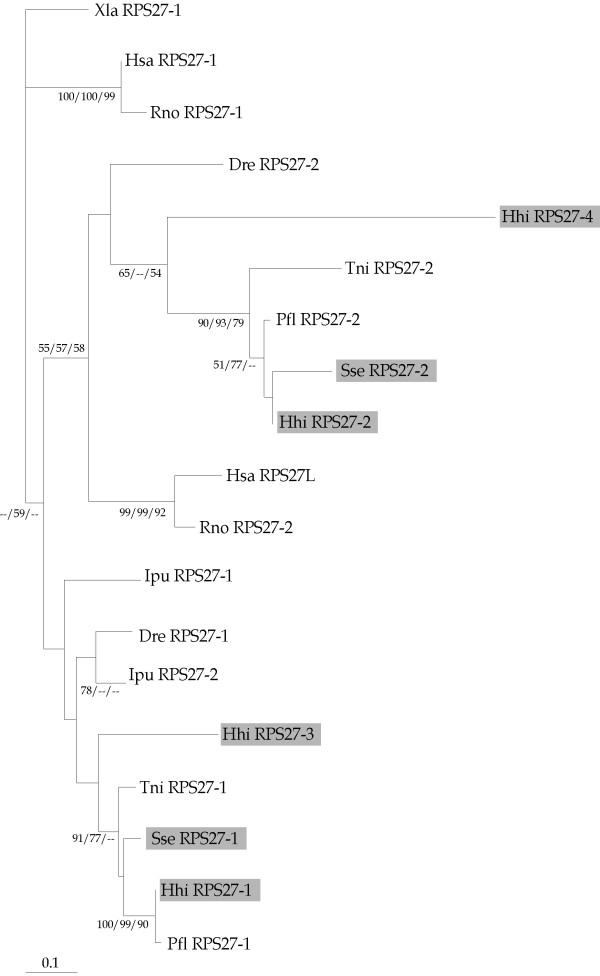
Phylogenetic relationships of RPS27 genes from *S. senegalensis *(Sse), *H. hippoglossus *(Hhi) *H. sapiens *(Hsa), *R. norvegicus *(Rno), *I. punctatus *(Ipu), *P. flesus *(Pfl), *D. rerio *(Dre) and *T. nigroviridis *(Tni). *Xenopus laevis *RPS27 was used as outgroup to root tree. Bootstrap values using NJ/MP/ML are indicated on each branch.

A phylogenetic analysis based on RPS27 coding sequences using the NJ, MP and ML methods showed that fish RPS27 genes grouped mainly in two distinct clades (Figure 2). Both Senegalese sole and Atlantic halibut RPS27-2 genes clustered with their fish counterparts. The RPS27-4 appeared more closely related to RPS27-2 than the other two Atlantic halibut RPS27 genes. Moreover, RPS27-2 and RPS27L genes in rat and human, respectively, formed a sister clade sharing a common ancestor with fish RPS27-2 genes (bootstrap values higher than 50%). The other fish RPS27 gene copies appeared linked in a clade that was not well resolved. Both Senegalese sole and Atlantic halibut RPS27-1 grouped with *P. flesus and T. nigroviridis *RPS27-1 (bootstrap values higher than 70%). Curiously, this clade contained both *I. punctatus *RPS27 genes.

### Gene expression analysis

We used a quantitative approach based on reverse transcription followed by real-time PCR amplification to investigate the steady-state levels of eight sole RP transcripts (RPS2, RPS3a, RPS15, RPS27-1, RPS27-2, RPS27a, RPS28, and RPS29) in liver, spleen, intestine, stomach, head kidney, gills, muscle, brain, heart, and skin. Relative gene expression levels were normalized by measuring ubiquitin levels.

**Figure 3:**
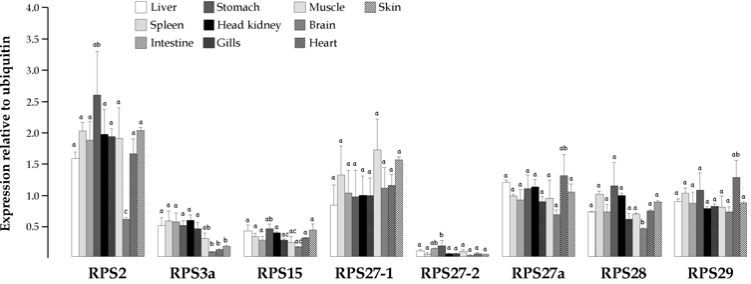
Gene expression of RPS2, RPS3a, RPS15, RPS27-1, RPS27-2 RPS27a, RPS28 and RPS29 in tissues from Senegalese sole. Expression ratios of each RP mRNA relative to ubiquitin mRNA ± SEM are shown. Values with the same superscript are not significantly different (*P *< 0.05).

All eight RP genes were expressed in detectable amounts in all tissues (Figure 3). RPS2 transcripts were the most abundant except in brain, where RPS27-1 showed the highest values (1.82-fold higher than RPS2). On the other hand, RPS27-2 was expressed at the lowest level in all tissues analyzed.

RP genes were expressed differentially among tissues. RPS2, RPS15, and RPS28 exhibited lower expression levels in brain and RPS3a transcripts were reduced in brain, heart, and skin. In contrast, RPS27-2 was expressed more highly in intestine and stomach, and RPS29 in heart. If we calculate the mean ribosomal expression ratio as a global RP expression index, all tissues showed similar values (0.74–0.99) except brain with only 0.48.

**Figure 4:**
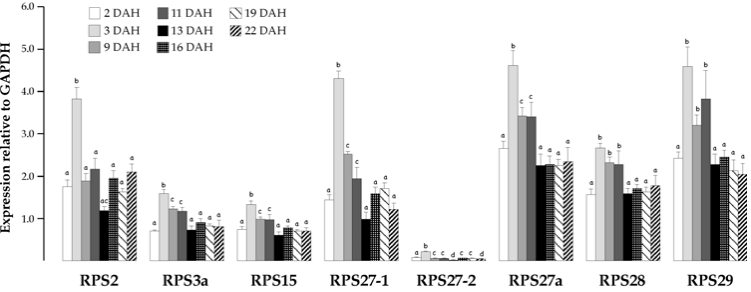
Gene expression of RPS2, RPS3a, RPS15, RPS27-1, RPS27-2 RPS27a, RPS28 and RPS29 during larval development in Senegalese sole. Expression ratios of each RP mRNA relative to GAPDH mRNA ± SEM are shown. Values with the same superscript are not significantly different (*P *< 0.05).

We also investigated the expression pattern of RP genes during sole larval development. mRNA levels were determined in samples extracted from whole larvae pools collected from 2 to 22 DAH (Figure 4). Expression levels of each RP gene were normalized to that of GAPDH. RPS2, RPS27-1, RPS27a, RPS28, and RPS29 showed higher transcript levels than RPS3a, RPS15 and RPS27-2 during early (2 to 3 DAH) larval development in Senegalese sole.

All RP mRNAs increased from 2 to 3 DAH, 24 hours after first external feeding (2.1-fold as global mean). Fold induction values ranged between 1.7 for both RPS27-1 and RPS28 and 3.0 for RPS27-2. These levels were reduced at 9 DAH, two days before the onset of eye migration, with the lowest values at the first metamorphic stages (13 DAH). The number of mRNA molecules for the 8 RPs analyzed declined approximately 3.5-fold as global mean from 3 (pre-metamorphosis) to 13 DAH (metamorphosis). The fold reduction values ranged between 1.7 and 10.3 for RPS28 and RPS27-2, respectively. RPS27-1 was expressed at a higher level than RPS27-2 during all pre-metamorphic, metamorphic and post-metamorphic stages (28-fold higher on average).

## Discussion

In this work, we describe the complete set of 40S RPs in the Senegalese sole and Atlantic halibut. Sequences were generated from normalized cDNA libraries constructed for expressed sequence tag (EST) analysis. The rapid development of genomics in all biological research areas, including aquaculture, and the high representation of RPs in the cDNA libraries have favoured the availability of an increasing number of RP sequences from different organisms [30]. This fact has motivated their proposed use as appropriate molecular markers for phylogenetic analysis. In fact, concatenation of orthologous RP amino acid sequences to form a single one of more than 10,000 characters has allowed the reconstruction of phylogenetic relationships between animal, fungal, and plant kingdoms [31]. With regard to this, Pleuronectiformes comprises a broad taxonomic group with 11 families and about 500 species worldwide, some of them of high commercially interest both in fisheries and aquaculture [32-34]. All flatfish species share in common an asymmetrical body development and a bottom-dwelling mode of life. However, their high phenotypic similarity has invoked great differences in the number and nomenclature of taxa depending on the relevance assigned to morphologic features [35-38]. Most phylogenetic studies focused on relationships among Pleuronectiformes have been based on partial mitochondrial DNA sequences [39-41]. The description of the complete set of RPs in one Pleuronectidae and Soleidae species provides new molecular markers to investigate the taxonomy and phylogeny among Pleuronectiformes. Also, the existence of paralogous genes exhibiting differential expression patterns in tissues, and even more important during larval development, particularly in metamorphosis, suggests RPs as interesting molecular markers to investigate flatfish genome evolution in terms of gain and loss of paralogous genes and the availabilty to acquire new functions (neofunctionalization) or divide the ancestral function between the paralogs (subfunctionalization) [42,43].

Three rounds of large-scale gene duplications (referred to as 1R, 2R, and 3R or fish-specific genome duplication) have been identified in fish [44,45]. These duplications are responsible, at least in part, for their speciation, adaptive radiation and high morphological complexity [45]. Although the majority of these gene duplicates have been lost or silenced during evolution, several gene copies have been described for some group of genes including glycolytic enzymes [44], Hox genes [46,47] and hormones and their receptors [48,49]. Similarly, different gene copies have been described for some RPs. In human, two different RPS4 genes exist, one encoded on the X and one on the Y chromosome. Rat possesses two distinct RPS27 transcripts that are expressed differentially in the hypothalamus [5]. In fish, *I. punctatus *has two paralogous genes of RPS26 and RPS27 [9]. In *S. senegalensis *and *H. hippoglossus*, two and four different RPS27 genes have been detected, respectively. Phylogenetic analysis revealed that RPS27-1 and RPS27-2 sequences grouped in two separate clades supported by significant bootstrap values. Although Thomas et al. [5] proposed the RPS27-2 as a mammalian-specific isoform, the identification of orthologous sequences for both RPS27 genes in different fish species supports the hypothesis of at least two RPS27 paralogs as a common feature in fish as well. Moreover, two additional RPS27 genes (referred to as RPS27-3 and RPS27-4) were identified in *H. hippoglossus*. These two paralogous genes might have appeared in the 3R or fish-specific genome duplication, In this respect, we should highlight that both RPS27 genes in *I. punctatus *grouped together in the same clade with *S. senegalensis *RPS27-1 and *H. hippoglossus *RPS27-1 and RPS27-3. This clustering suggests the existence of, at least, a third RPS27 gene in *I. punctatus *orthologous to fish RPS27-2. Such a hypothesis is also supported by the fact that both *I. punctatus *RPS27 paralogs were expressed at a similar level (represented by 7 and 10 clones for RPS27-1 and RPS27-2, respectively) [9], whereas in *S. senegalensis *RPS27-2 was expressed at a much lower level than RPS27-1 in all tissues and during larval development as determined by real-time PCR.

During embryogenesis, after mid-blastula transition in zebrafish, RP genes co-ordinately increase their expression [16-18]. In addition, in Atlantic halibut up to 40 and 41 RPs increase mRNA levels from embryos to 1 day-old yolk sac larvae and fast skeletal muscle in juveniles, respectively [15]. In this study, we provide evidence that one day after first feeding, the eight RPs analyzed by real-time PCR increase their expression levels in Senegalese sole also. During this period, larvae undergo important physiological and morphological changes such as the opening of the mouth and anus. When live prey are provided for feeding, different organs such as the liver, pancreas, and the digestive tract are activated, promoting larval metabolism [50]. Larval rearing is a critical period during which different aspects concerning important anatomical and physiological traits in the juvenile stage are modulated. There are reports on biomarkers for fish larvae fed different diets that focused specifically on oxidative stress [51] and digestive enzymes [52]. The co-ordinate changes in RP expression under important physiological events such as the first feeding suggest RPs might be considered as biomarkers that could provide broader information about the general physiological condition in fish. In addition, the high abundance of these RPs (about 50% of RNA polymerase II transcription in rapidly growing yeast cells [53]), most of which are considered as house-keeping genes, indicates that even small induction values, as observed in this survey (1.7–3.0 fold), can play an important physiological role.

Although RP transcript levels increased after first feeding, they dropped at the first metamorphic stages (13 DAH). In this respect, we should take into account that Senegalese sole exhibits two different growth rates during larval development. At pre-metamorphosis, larvae grow at almost twice the rate as at metamorphosis and accumulate energy reserves in tissues to be used during this important period [26,29]. The lower growth rate at metamorphosis has been correlated with reduced IGF-II expression levels [54], an activator of the 70-kDa ribosomal S6 kinase (S6K1), a serine/threonine protein kinase that plays a central role in cell growth and proliferation. This kinase mediates the phosphorylation of RPS6, thereby enabling efficient translation of 5'-terminal oligopyrimidine tract (5'-TOP) mRNAs. Since RPs and translation elongation factors are encoded by 5'-TOP mRNAs, signalling along the S6K1 pathway may regulate ribosome biogenesis and therefore the response to growth conditions [55,56]. Moreover, apoptosis has been shown to play an important role in the organ-rebuilding process during flatfish metamorphosis [57] and some RPs have been associated with apoptotic processes [25,58]. The reduction in RP gene expression, especially at the beginning of sole metamorphosis, suggests they could also be involved in the control of apoptosis during metamorphosis.

We evaluated gene expression of eight RPs in ten different sole tissues. Overall, all tissues except brain expressed RPs at a similar level. These data agree with those obtained in *I. punctatus *using a transcriptomic approach. Representation of RPs was reduced in brain compared with skin and head kidney [9]. Ribosome formation can vary in response to cellular demands and their protein biosynthetic requirements [59] and these differences in the steady-state number of RP transcripts might reflect the distinct metabolic activity of tissues.

RPs exhibited different expression levels in different sole tissues. RPS27-2 mRNA levels were up to 41.9 and 54.7-fold lower than RPS27-1 and RPS2, respectively. These differences in relative mRNA abundance among RPs were also observed in *I. punctatus *and *H. hippoglossus *larvae and juveniles [9,15]. Such difference suggests a translational regulation to facilitate the correct assembly of ribosomes. Moreover, there is increasing evidence that RPs modulate a variety of cellular activities independent of their own involvement in the protein biosynthesis such as replication, transcription, RNA processing, DNA repair, and inflammation [60]. In our study, some RPs exhibited different tissue expression patterns. For instance, RPS2 was highly expressed in all tissues except in brain where RPS27-1 transcripts were the highest. These data agree with those described for *I. punctatus *where the number of ESTs corresponding to RPS2 and RPS27 were 10-fold lower and 3-fold higher, respectively, in brain than in skin and head kidney [9]. In addition, RPS3a transcripts were reduced in brain, heart, and skin, and RPS27-2 showed the highest expression levels in intestine and stomach. All these data underscore the necessity for new studies to elucidate the regulation of these RPs in tissues and their possible extraribosomal function.

## Conclusion

In this work we have identified and characterized the complete set of 40S RPs in two Pleuronectiformes: Senegalese sole and Atlantic halibut. These data provide new molecular markers to investigate genome evolution and phylogenetic relationships among flatfish. Also, gene expression studies in Senegalese sole have revealed a co-ordinate response after first feeding in larvae suggesting a possible role of RPs as general condition biomarkers to estimate larval physiological status in response to changing environmental conditions. Moreover, the differential expression patterns in tissues suggest that RPs might perform other functions distinct from protein biosynthesis.

## Methods

### Identification of RP cDNAs in Senegalese sole and Atlantic halibut

Ten cDNA libraries were constructed from different larval stages and adult tissues of Senegalese sole using the ZAP Express^® ^cDNA Syntesis kit and Zap Express cDNA Gigapack^® ^III Gold Cloning kit (Stratagene) following the manufacturer's protocol (Cerdà et al., in preparation). The libraries were pooled and normalized, and approximately 11,000 randomly selected clones were sequenced from the 3'-end. Expressed sequence tags (ESTs) encoding RPs were identified after EST annotation. For RPS28 isolation, we designed specific primers (Table 1) using a partial sequence from a suppression subtractive hybridization library. RPS28 was amplified from the premetamorphic larval development library using combination of specific primers and the universal primers T3 and T7.

In halibut, normalised cDNA libraries were constructed for five different larval time points (hatching, mouth-opening, midway to metamorphosis, premetamorphosis, and postmetamorphosis) and eight adult tissues (testis, ovary, liver, head kidney, spleen, skin, gill, and intestine) [61], incorporated into the Pleurogene database  and provisionally annotated using AUTOFACT [62] implemented on the database.

### Fish sampling

Juvenile Senegalese sole individuals (n = 3) were obtained from IFAPA Centro *El Toruño *facilities (El Puerto Santa María, Cádiz, Spain). They were sacrificed by immersion in tricaine methanesulfonate (MS-222). Liver, spleen, intestine, stomach, head kidney, gills, muscle, brain, heart, and skin were rapidly dissected, frozen in liquid nitrogen and stored at -80°C until use.

For larval studies, fertilized eggs from a naturally spawning Senegalese sole broodstock (IFAPA Centro *El Toruño*) were collected. They were incubated in a 150 L tank at 19–21°C for two days. Newly hatched larvae were transferred to a 400 L tank at an initial density from 45 to 50 larvae L^-1 ^with a 16L:8D photoperiod and a light intensity of 600–800 lux. Larvae were fed rotifers (*Brachionus plicatilis*) 3 DAH till 9 DAH. From 7 DAH enriched artemia metanauplii were fed until the end of the experiment. Pools of larvae from 2 to 22 DAH (n = 3) were collected, washed with DEPC water, frozen in liquid nitrogen and stored at -80°C until analysis.

### RNA isolation and gene expression analysis

Homogenization of juvenile tissues and larvae was carried out using Lysing Matrix D (Q-BioGene) for 40 s at speed setting 6 in the Fastprep FG120 instrument (Bio101). Total RNA was isolated from 50 mg of *S. senegalensis *tissues or pools of larvae using the RNeasy Mini Kit (Qiagen). All RNA isolation procedures were performed in accordance with the manufacturer's protocol. In all cases, total RNA was treated twice with DNase I using the RNase-Free DNase kit (Qiagen) for 30 min in order to avoid amplification of contaminated genomic DNA. RNA sample quality was checked using Experion (Bio-Rad) and quantification was performed spectrophotometrically. Total RNA (1 μg) from each sample was reverse-transcribed using the iScript™ cDNA Synthesis kit (Bio-Rad). Reverse transcription reactions were performed in duplicate. Lack of genomic DNA contamination was confirmed by PCR amplification of RNA samples in the absence of cDNA synthesis.

Real-time analysis was carried out on an iCycler (Bio-Rad). Reactions were performed in a 25 μl volume containing cDNA generated from 10 ng of original RNA template, 300 nM each of specific forward (F) and reverse (R) primers (Table 1), and 12.5 μl of iQ™ SYBR Green Supermix (Bio-Rad). Matching oligonucleotide primers were designed using Oligo *v*6.89 software (Medprobe). The amplification protocol used was as follows: initial 7 min denaturation and enzyme activation at 95°C, 40 cycles of 95°C for 15 s and 70°C for 30 s. Each assay was performed in duplicate. For normalization of cDNA loading, all samples were run in parallel with a housekeeping gene (glyceraldehyde-3-phosphate dehydrogenase (GAPDH; [DDBJ:AB291587]) or ubiquitin ([DDBJ:AB291588]) for larval development or juvenile tissues, respectively). To estimate efficiencies, a standard curve was generated for each primer pair based on known quantities of cDNA (10-fold serial dilutions corresponding to cDNA transcribed from 100 to 0.01 ng of total RNA). All calibration curves exhibited correlation coefficients higher than 0.99 and the corresponding real-time PCR efficiencies were 0.90–0.95. Relative mRNA expression of RPs was determined using the Ct method (value obtained by subtracting the Ct value of GAPDH or ubiquitin mRNA from the Ct value of the target mRNA). Data was expressed as the ratio (calculated using 1.93^-(ΔCt)^) of target mRNA to reference (GAPDH or ubiquitin) mRNA. Owing to the small differences among amplicons both in size (87–139 bp) and %GC (45.4–65.4), the relative sensitivity factor K_RS _was assumed to be one.

Results were expressed as mean ± SEM. Comparisons among groups were performed with one-way analysis of variance, followed by a Tukey test for identification of the statistically distinct groups. Significance was accepted for *P *< 0.05.

### Sequence and phylogenetic analysis

Alignments of sequences were carried out and the sequence similarities calculated by the MegAlign program from the LASERGENE software suite. For phylogenetic analysis, sequences of RPS27 from different species including *Homo sapiens *([GenBank:HSU57847, GenBank:NM_015920]; [6,63]), *Rattus norvegicus *([GenBank:AF184893, EMBL:X59375]; [5,64]), *Xenopus laevis *([GenBank:BC053815]; [65]), *Ictalurus punctatus *([GenBank:AF402836, GenBank:AF402837]; [9]), *Platichthys flesus *([GenBank:DV566302 and GenBank:DV567451]; unpublished), *Danio rerio *([GenBank:BQ077524, GenBank:BC114281]; unpublished) and *Tetraodon nigroviridis *([EMBL:CR722207 and EMBL:CR642405]; unpublished) were employed. Coding sequences were aligned using MegAlign software. Neighbor-joining (NJ), maximum parsimony (MP) and maximum likelihood (ML) analyses were carried out using PAUP*v*4*beta*10 software [66]. *The TrNef + G model *of sequence evolution was the most appropriate as selected by MODELTEST *v*3.5 [67]. The parameters of ML methods were R(a) = 1.0000, R(b) = 3.2865, R(c) = 1.0000, R(d) = 1.0000, and R(e) = 6.057. The gamma distribution shape parameter was estimated to be 0.3234. The degree of confidence assigned to nodes in trees was achieved by bootstrapping with 1,000 replicates.

## Authors' contributions

MM designed the study, carried out the phylogenetic analyses, and drafted the manuscript. CI carried out the gene expression analysis and helped to draft the manuscript. EA performed the Senegalese sole cultures and samplings. JPC participated in the study design and coordination and helped to draft the manuscript. SED participated in sequence analysis and drafted the manuscript. All authors read and approved the final manuscript.

## References

[B1] Zarivach R, Bashan A, Berisio R, Harms J, Auerbach T, Schluenzen F, Bartels H, Baram D, Pyetan E, Sittner A (2004). Functional aspects of ribosomal architecture: symmetry, chirality and regulation. J Phys Org Chem.

[B2] Wool IG, Endo Y, Chan YL, Glück A, Hill W, Dahlberg A, Garrett R, Moore P, Schlessinger D, Warner J (1990). Studies of the structure, function and evolution of mammalian ribosomes. Ribosome Structure, Function and Evolution.

[B3] Wool IG, Chan YL, Glück A (1995). Structure and evolution of mammalian ribosomal proteins. Biochem Cell Biol.

[B4] Fisher EM, Beer-Romero P, Brown LG, Ridley A, McNeil JA, Lawrence JB, Willard HF, Bieber FR, Page DC (1990). Homologous ribosomal protein genes on the human X and Y chromosomes: escape from X inactivation and possible implications for Turner syndrome. Cell.

[B5] Thomas EA, Alvarez CE, Sutcliffe JG (2000). Evolutionarily distinct classes of S27 ribosomal proteins with differential mRNA expression in rat hypothalamus. J Neurochem.

[B6] He H, Sun Y (2007). Ribosomal protein S27L is a direct p53 target that regulates apoptosis. Oncogene.

[B7] Planta RJ, Mager WH (1998). The list of cytoplasmic ribosomal proteins of *Saccharomyces cerevisiae*. Yeast.

[B8] Ribosomal Protein Gene Database. http://ribosome.med.miyazaki-u.ac.jp.

[B9] Karsi A, Patterson A, Feng J, Liu Z (2002). Translational machinery of channel catfish: I. A transcriptomic approach to the analysis of 32 40S ribosomal protein genes and their expression. Gene.

[B10] Patterson A, Karsi A, Feng J, Liu Z (2003). Translational machinery of channel catfish: II. Complementary DNA and expression of the complete set of 47 60S ribosomal proteins. Gene.

[B11] Cujec TP, Tyler BM (1996). Nutritional and growth control of ribosomal protein mRNA and rRNA in *Neurospora crassa*. Nucleic Acids Res.

[B12] Herruer MH, Mager WH, Woudt LP, Nieuwint RT, Wassenaar GM, Groeneveld P, Planta RJ (1987). Transcriptional control of yeast ribosomal protein synthesis during carbon-source upshift. Nucleic Acids Res.

[B13] Milne AN, Mak WW, Wong JT (1975). Variation of ribosomal proteins with bacterial growth rate. J Bacteriol.

[B14] Waldron C, Jund R, Lacroute F (1977). Evidence for a high proportion of inactive ribosomes in slow-growing yeast cells. Biochem J.

[B15] Bai J, Solberg C, Fernandes JM, Johnston IA (2006). Profiling of maternal and developmental-stage specific mRNA transcripts in Atlantic halibut *Hippoglossus hippoglossus*. Gene.

[B16] Linney E, Dobbs-McAuliffe B, Sajadi H, Malek RL (2004). Microarray gene expression profiling during the segmentation phase of zebrafish development. Comp Biochem Physiol C Toxicol Pharmacol.

[B17] Lo J, Lee S, Xu M, Liu F, Ruan H, Eun A, He Y, Ma W, Wang W, Wen Z (2003). 15000 unique zebrafish EST clusters and their future use in microarray for profiling gene expression patterns during embryogenesis. Genome Res.

[B18] Mathavan S, Lee SG, Mak A, Miller LD, Murthy KR, Govindarajan KR, Tong Y, Wu YL, Lam SH, Yang H (2005). Transcriptome analysis of zebrafish embryogenesis using microarrays. PLoS Genet.

[B19] Kowalczyk P, Woszczynski M, Ostrowski J (2002). Increased expression of ribosomal protein S2 in liver tumors, posthepactomized livers, and proliferating hepatocytes *in vitro*. Acta Biochim Pol.

[B20] Pogue-Geile K, Geiser JR, Shu M, Miller C, Wool IG, Meisler AI, Pipas JM (1991). Ribosomal protein genes are overexpressed in colorectal cancer: isolation of a cDNA clone encoding the human S3 ribosomal protein. Mol Cell Biol.

[B21] Cramton SE, Laski FA (1994). String of pearls encodes Drosophila ribosomal protein S2, has Minute-like characteristics, and is required during oogenesis. Genetics.

[B22] Watson KL, Konrad KD, Woods DF, Bryant PJ (1992). Drosophila homolog of the human S6 ribosomal protein is required for tumor suppression in the hematopoietic system. Proc Natl Acad Sci USA.

[B23] Kim J, Chubatsu LS, Admon A, Stahl J, Fellous R, Linn S (1995). Implication of mammalian ribosomal protein S3 in the processing of DNA damage. J Biol Chem.

[B24] Draptchinskaia N, Gustavsson P, Andersson B, Pettersson M, Willig TN, Dianzani I, Ball S, Tchernia G, Klar J, Matsson H (1999). The gene encoding ribosomal protein S19 is mutated in Diamond-Blackfan anaemia. Nat Genet.

[B25] Naora H, Takai I, Adachi M, Naora H (1998). Altered cellular responses by varying expression of a ribosomal protein gene: sequential coordination of enhancement and suppression of ribosomal protein S3a gene expression induces apoptosis. J Cell Biol.

[B26] Fernández-Díaz C, Yúfera M, Cañavate JP, Moyano FJ, Alarcón FJ, Díaz M (2001). Growth and physiological changes during metamorphosis of Senegal sole reared in the laboratory. J Fish Biol.

[B27] Haug T (1990). Biology of the Atlantic halibut, *Hippoglossus hippoglossus *(L. 1758). Adv Mar Biol.

[B28] Power DM, Llewellyn L, Faustino M, Nowell MA, Bjornsson BT, Einarsdottir IE, Canario AV, Sweeney GE (2001). Thyroid hormones in growth and development of fish. Comp Biochem Physiol C Toxicol Pharmacol.

[B29] Parra G, Yúfera M (2001). Comparative energetics during early development of two marine fish species, *Solea senegalensis *(KAUP) and *Sparus aurata *(L.). J Exp Biol.

[B30] Nakao A, Yoshihama M, Kenmochi N (2004). RPG: the Ribosomal Protein Gene database. Nucleic Acids Res.

[B31] Veuthey AL, Bittar G (1998). Phylogenetic relationships of fungi, plantae, and animalia inferred from homologous comparison of ribosomal proteins. J Mol Evol.

[B32] Nelson JS (1994). Fishes of the world.

[B33] Helfman G, Collette B, Facey D (1997). The diversity of fishes.

[B34] Froese R, Pauly D (2007). FishBase. World Wide Web electronic publication version (01/2007).

[B35] Chapleau F (1993). Pleuronectiform relationships: a cladistic reassessment. Bull Mar Sci.

[B36] Cooper JA, Chapleau F (1998). Monophyly and intrarelationships of the family Pleuronectidae (Pleuronectiformes), with a revised classification. Fish Bull.

[B37] Hensley DA (1997). An overview of the systematics and biogeography of the flatfishes. J Sea Res.

[B38] Hoshino K (2001). Monophyly of the Citharidae (Pleuronectoidei: Pleuronectiformes: Teleostei) with considerations of pleuronectoid phylogeny. Ichthyol Res.

[B39] Pardo BG, Machordom A, Foresti F, Porto-Foresti F, Azevedo MFC, Bañón R, Sánchez L, Martínez P (2005). Phylogenetic analysis of flatfish (Order Pleuronectiformes) based on mitochondrial 16S rDNA sequences. Sci Mar.

[B40] Infante C, Catanese G, Manchado M (2004). Phylogenetic relationships among ten sole species (Soleidae, Pleuronectiformes) from the Gulf of Cadiz (Spain) based on mitochondrial DNA sequences. Mar Biotechnol (NY).

[B41] Berendzen PB, Dimmick WW (2002). Phylogenetic relationships of Pleuronectiformes based on molecular evidence. Copeia.

[B42] Force A, Lynch M, Pickett FB, Amores A, Yan YL, Postlethwait J (1999). Preservation of duplicate genes by complementary, degenerative mutations. Genetics.

[B43] Sidow A (1996). Gen(om)e duplications in the evolution of early vertebrates. Curr Opin Genet Dev.

[B44] Steinke D, Hoegg S, Brinkmann H, Meyer A (2006). Three rounds (1R/2R/3R) of genome duplications and the evolution of the glycolytic pathway in vertebrates. BMC Biol.

[B45] Volff JN (2005). Genome evolution and biodiversity in teleost fish. Heredity.

[B46] Hoegg S, Brinkmann H, Taylor JS, Meyer A (2004). Phylogenetic timing of the fish-specific genome duplication correlates with the diversification of teleost fish. J Mol Evol.

[B47] Hoegg S, Meyer A (2005). Hox clusters as models for vertebrate genome evolution. Trends Genet.

[B48] Moncaut N, Somoza G, Power DM, Canario AV (2005). Five gonadotrophin-releasing hormone receptors in a teleost fish: isolation, tissue distribution and phylogenetic relationships. J Mol Endocrinol.

[B49] Sherwood NM, Adams BA, Melamed P, Sherwood NM (2005). Gonadotropin-Releasing hormone in fish: Evolution, expression and regulation of the GnRH gene. Hormones and Their Receptors in Fish Reproduction.

[B50] Ribeiro L, Sarasquete C, Dinis MT (1999). Histological and histochemical development of the digestive system of *Solea senegalensis *(Kaup, 1858) larvae. Aquaculture.

[B51] Fernández-Díaz C, Kopecka J, Cañavate JP, Sarasquete C, Solé M (2006). Variations on development and stress defences in *Solea senegalensis *larvae fed on live and microencapsulated diets. Aquaculture.

[B52] Gudmundsdottir A, Palsdottir HM (2005). Atlantic cod trypsins: from basic research to practical applications. Mar Biotechnol (NY).

[B53] Warner JR (1999). The economics of ribosome biosynthesis in yeast. Trends Biochem Sci.

[B54] Funes V, Asensio E, Ponce M, Infante C, Cañavate JP, Manchado M (2006). Insulin-like growth factors I and II in the sole *Solea senegalensis*: cDNA cloning and quantitation of gene expression in tissues and during larval development. Gen Comp Endocrinol.

[B55] Jefferies HB, Fumagalli S, Dennis PB, Reinhard C, Pearson RB, Thomas G (1997). Rapamycin suppresses 5'TOP mRNA translation through inhibition of p70s6k. EMBO J.

[B56] Terada N, Patel HR, Takase K, Kohno K, Nairn AC, Gelfand EW (1994). Rapamycin selectively inhibits translation of mRNAs encoding elongation factors and ribosomal proteins. Proc Natl Acad Sci USA.

[B57] Bao-Long B, Gui-Mei Y, Da-Ming R (2006). Apoptosis in the metamorphosis of Japanese flounder *Paralichthys olivaceus*. Acta Zool Sin.

[B58] Bushell M, Stoneley M, Sarnow P, Willis AE (2004). Translation inhibition during the induction of apoptosis: RNA or protein degradation?. Biochem Soc Trans.

[B59] Woolford JL, Warner JR, Broach JR, Pringle JR, Jones EW (1991). The ribosome and its synthesis. The Molecular Biology and Cellular Biology of the Yeast Saccharomyces cerevisiae.

[B60] Wool IG (1996). Extraribosomal functions of ribosomal proteins. Trends Biochem Sci.

[B61] Douglas SE, Knickle LC, Kimball J, Reith ME Comprehensive EST analysis of Atlantic halibut (*Hippoglossus hippoglossus*), a commercially relevant aquaculture species. BMC genomics.

[B62] Koski LB, Gray MW, Lang BF, Burger G (2005). AutoFACT: an automatic functional annotation and classification tool. BMC Bioinformatics.

[B63] Tsui SK, Lee SM, Fung KP, Waye MM, Lee CY (1996). Primary structures and sequence analysis of human ribosomal proteins L39 and S27. Biochem Mol Biol Int.

[B64] Chan YL, Suzuki K, Olvera J, Wool IG (1993). Zinc finger-like motifs in rat ribosomal proteins S27 and S29. Nucleic Acids Res.

[B65] Klein SL, Strausberg RL, Wagner L, Pontius J, Clifton SW, Richardson P (2002). Genetic and genomic tools for Xenopus research: The NIH Xenopus initiative. Dev Dyn.

[B66] Swofford DL (2003). PAUP*. Phylogenetic Analysis Using Parsimony (*and Other Methods).

[B67] Posada D, Crandall KA (1998). Modeltest: testing the model of DNA substitution. Bioinformatics.

